# Integrated metagenomics and targeted-metabolomics analysis of the effects of phenylalanine on loperamide-induced constipation in rats

**DOI:** 10.3389/fmicb.2022.1018008

**Published:** 2022-09-30

**Authors:** Chuanli Yang, Xinshu Bai, Tianjiao Hu, Xin Xue, Xiaohu Su, Xuan Zhang, Teng Wu, Mingxia Zhang, Xiaobing Shen, Xiushan Dong

**Affiliations:** ^1^Key Laboratory of Environmental Medical Engineering and Education Ministry, School of Public Health, Southeast University, Nanjing, Jiangsu, China; ^2^Department of Preventive Medicine, School of Public Health, Southeast University, Nanjing, Jiangsu, China; ^3^Department of General Surgery, Shanxi Bethune Hospital, Shanxi Academy of Medical Sciences, Tongji Shanxi Hospital, Third Hospital of Shanxi Medical University, Taiyuan, Shanxi, China; ^4^Department of Clinical Laboratory, Shanxi Bethune Hospital, Shanxi Academy of Medical Sciences, Tongji Shanxi Hospital, Third Hospital of Shanxi Medical University, Taiyuan, Shanxi, China

**Keywords:** phenylalanine, constipation, gut microbiota, metagenomics, metabolomics, neurotransmitter

## Abstract

Functional constipation is a common functional gastrointestinal disease. In our previous study, we found that the gut microbiota structure was disordered and the level of phenylalanine (Phe) in serum was decreased in constipated women. We conducted the present study to elucidate the role of Phe in remodeling the composition of gut microbiota and the relationship between gut microbiota and serum metabolites. Here, we demonstrated that Phe treatment significantly enhanced intestinal motility, suppressed inflammatory responses, and prevented intestinal barrier damage in rats with loperamide (Lop)-induced constipation. By metagenomic sequencing, the disbalanced gut microbial profile was analyzed in constipated rats. Phe treatment reversed changes in the abundance of several gut bacteria at the phylum, genus, and species levels. Further, we observed distinct metabolic patterns in constipated rats through targeted metabolomics and identified constipation-related gut microbial species linked to changes in circulating neurotransmitter metabolites. The abundances of species *s_Lactobacillus murinus*, *s_Enterococcus italicus*, *s_Lactobacillus animalis*, *s_Lactobacillus apodemi*, *s_Enterococcus faecalis*, and *s_Lactobacillus backii* were positively correlated with L-asparagine, L-Glutamic acid, Putrescine, and Spermidine levels. The abundances of *s_Lactobacillus johnsonii* and *s_Butyricimonas virosa* were negatively correlated with L-asparagine, L-Glutamic acid, Putrescine, and Spermidine levels. Taken together, our findings suggest that Phe can ameliorate the development of Lop-induced constipation in rats by remodeling the gut microbial community structure and changing metabolite levels.

## Introduction

Functional gastrointestinal diseases, also termed disorders of gut–brain interaction, are a common diagnosis in gastroenterology (Black et al., 2020). They frequently occur in combination with morphological and physiological abnormalities, including motility disturbance, visceral hypersensitivity, altered mucosal and immune function, altered gut microbiota, and altered central nervous system processing (Drossman, 2016). They lead to higher global healthcare costs (Tack et al., 2019) and reduce health-related quality of life (Wong and Drossman, 2010). According to the newly revised Rome IV standard, chronic functional constipation was defined as a disorder of intestinal brain function interaction (Drossman and Hasler, 2016). Moreover, a number of clinical studies have shown that anxiety and depression were present in patients with functional constipation (Albiani et al., 2013; Bouchoucha et al., 2014). Although the etiology of functional constipation is still unclear, the currently accepted theory is the “microbe-gut-brain axis.” Therefore, it is particularly important to explore the relationship between gut microbiota and neurotransmitters in functional constipation.

The diet, central nervous system, gut microbiota, and substances derived from the gut microbiota form a complex bidirectional network known as the brain-gut-microbiome axis, which is influenced by natural and social environments (Martin et al., 2018). If any level of this axis were disturbed, the homeostasis of the body would be disturbed, which may lead to the development of undesirable diseases, such as gastrointestinal and hepatic diseases, metabolic diseases, cardiovascular diseases, immune-related diseases, oncologic diseases, and neurologic and psychiatric diseases (Gomaa, 2020). An increasing body of proof from humans and animals suggests that there is a powerful correlation between gut microbiota and constipation based on the brain–gut–microbiome axis (Park et al., 2013; Jacobs et al., 2021; Zhang et al., 2021). Microbial treatment can alleviate the clinical symptoms of patients with functional constipation, including defecation frequency and stool consistence (Choi and Chang, 2015; Ge et al., 2016). Once we consider that the gut microbiota and their metabolites serve as a “transfer station” on this axis, it is crucial to investigate the explicit microbial mechanisms involved in disease development.

The abundance of *g_Bacteroides* was found to be significantly increased in constipation patients of reproductive age by 16S RNA sequencing (Li et al., 2020), but Khalif et al., (2005), who used culturing methods (mean age 42.2 years), found that the abundance of *g_Bacteroides* was decreased in patients with chronic constipation. Kim et al. used the real-time polymerase chain reaction (PCR) method and found that the abundance of *g_Bacteroides* was decreased in patients with functional constipation (mean age, 35 years; Kim et al., 2015). This discrepancy may be due to the influence of confounding factors such as age, gender, and detection methods, which lead to inconsistent or even contradictory research results. Therefore, it is necessary to consider relevant confounding factors and adopt a multi-omics research strategy to draw more reliable conclusions.

Phenylalanine (Phe) is a precursor to catecholamines (such as dopamine, norepinephrine, and epinephrine) and is essential for the biosynthesis of these neurotransmitters (Fernstrorn and Fernstrom, 2007). Multiple studies have found that Phe metabolism disorder was related to dementia, which may be due to the influence on the balance of neurotransmitters in the brain (Wissmann et al., 2013; Liu P. et al., 2021). In addition, gray matter and white matter changes were observed in the brain of IBS patients, including somatosensory systems such as thalamus and basal ganglia (Mayer et al., 2015). Furthermore, a study on aging and constipation found that both constipated and aging rats exhibited constipation and aging phenotypes, and through fecal metabolomics, it was found that there were differences in the metabolic profiles between the two groups, in which the level of Phe was increased in the constipation group, but it was decreased in the aging group (Liu X. et al., 2021). In our previous study, it was shown that Phe levels decreased in female patients of reproductive age with constipation, and the Phe metabolic pathway was abnormal (Liu et al., 2022). Thus, Phe may play an essential character in the pathophysiological process of chronic constipation, but the molecular mechanism is still unclear.

Therefore, in this study, we used female rats with loperamide (Lop)-induced constipation and intervened with Phe. We used metagenomics and metabolomics technology to analyze the role of Phe in the pathophysiological process of constipation and the potential mechanism.

## Materials and methods

### Reagents

Lop, L-Phe, active charcoal, and carboxymethylcellulose were purchased from Sigma-Aldrich (St. Louis, MO, United States). Hematoxylin and eosin staining kit was purchased from Beyotime (Shanghai, China).

### Lop-induced constipation model

**Figure 1:**
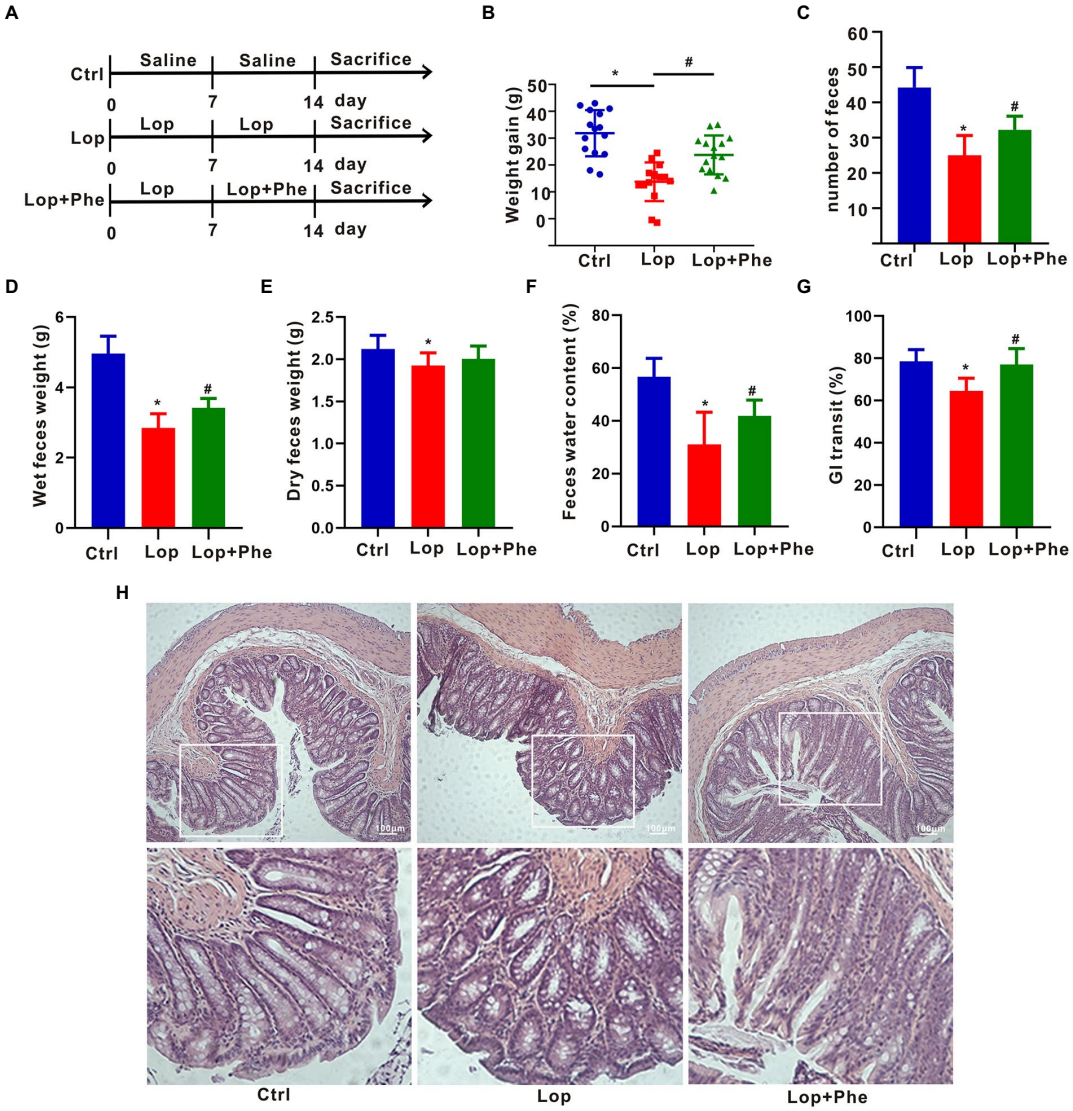
Effects of Phe on defecation-related parameters and colonic tissue injury in constipated rats. **(A)** Experimental process. **(B)** Weight gain (*n* = 15). **(C)** Number of feces (*n* = 5). **(D)** Weight of wet feces (*n* = 5). **(E)** Weight of dry feces (*n* = 5). **(F)** Fecal water content (*n* = 5). **(G)** Gastrointestinal transit rate (*n* = 3). **(H)** Colonic staining using hematoxylin and eosin (*n* = 3). *p < 0.05 compared to Ctrl group; #*p* < 0.05 compared to LOP group.

Female 8-week-old Sprague Dawley rats were purchased from Beijing Vital River Laboratory Animal Technology (Beijing, China). Before the experiment, the animals were housed in a room at a temperature of 20–22°C and relative humidity of 60–70% under a 12/12 h light/dark cycle. The animals were randomly divided into three groups, with 15 animals in each group: the control (Ctrl) group, the Lop group, and the Lop + Phe group. As shown in Figure 1A, the Ctrl group was administered saline solution, and the Lop group was conducted 10 mg/kg Lop solution for 14 days. The Lop + Phe group was conducted 10 mg/kg Lop solution in the first week, and followed by the gavage with 10 mg/kg Lop and 25 mg/kg Phe solution in combination in the second week. All solutions were administered by gavage once a day for 14 days. All rats were killed at the end of treatment. The animal experiments were approved by the Animal Experiment Committee of the China Institute for Radiation Protection and were carried out according to the animal experiment guidelines of the China Institute for Radiation Protection.

### Body weight and fecal parameters

Body weight was assessed weekly throughout the experimental period. In order to take differences in baseline weight into account, we calculated the weight increase in each group. After 2 weeks of Lop induction, fresh fecal pellets were gathered from each rat within 24 h. The collected fecal pellets were removed moisture in a forced ventilation oven at 60°C until the weight was constant. The fecal water content was estimated depending on the following formula:

Fecal water content (%) = [(wet weight − dry weight)/wet weight] × 100%.

### Intestinal motility assay

Intestinal motility was estimated on the basis of the method described by Li *et al* (Li et al., 2015). Briefly, at the end of the 2-week intervention, the rats (*n* = 5 per group) were fasted for 12 h. Carbon solution was processed by dissolving 10% charcoal powder in 0.5% carboxymethylcellulose solution; the rats were administered the prepared carbon solution by gavage, and 30 min later, the rats were killed. The whole intestine of each rat was gathered to evaluate the distance of carbon movement. The formula below was used to calculate the transit ratio of intestinal movement:

Intestinal transit ratio (%) = distance of carbon powder movement/full length of small intestine × 100%.

### Hematoxylin–eosin staining

Fresh colon tissues were fixed with 4% paraformaldehyde, dehydrated, paraffin-embedded, cut into 5-μm-thick slices, and stained with a hematoxylin and eosin staining kit (Beyotime, Shanghai, China). The histomorphological changes were observed by using a Zeiss (Germany) microscope.

### Fecal DNA isolation, metagenomic sequencing, and analysis

The rat gut microbiome was investigated using a metagenomic shotgun sequencing approach as described previously (Zhang et al., 2020). Genome DNA was extracted from 22 fecal samples as previously described. Agarose gel electrophoresis was employed to assess the integrity and purity of DNA. Certified DNA samples were randomly broken into 350 bp fragments with a Covaris ultrasonic breaker, and the entire library was established through end repair, A-tail addition, adapter ligation, purification, and amplification. After the library was constructed, for purpose of ensuring the quality of the library, the insert size of the library was determined using an Agilent 2,100, and the appropriate concentration of the library was detected by qRT-PCR. Then, the metagenomic sequencing was performed on an Illumina PE150 sequencing platform at Shanghai Biotree Biomedical Technology (Shanghai, China) according to the manufacturer’s instructions. The raw data obtained by sequencing were subjected to quality control to acquire pure data. Pure data were assembled by metagenome, and MetaGeneMark is a common software used for gene prediction. Analyzed data were compared with the MicroNR library to get the species annotation information of UniGene. Kyoto Encyclopedia of Genes and Genomes (KEGG) metabolic pathway function annotation and abundance analysis was carried out, and cluster analysis, principal component analysis (PCA), and analysis of similarities (ANOSIM) were conducted based on species abundance.

### Targeted metabonomic analysis based on UHPLC–MS/MS

A total of 30 serum samples from three groups were subjected to targeted metabolomic analysis. Each individual sample, 20 μl was transferred to a sterile Eppendorf tube. Then add 80 μl of extract solvent (acetonitrile with 0.1 percent formic acid, pre-cooled at −20°) to the above sample, the samples were fully vortexed for 30 s and sonicated in an ice water bath for 15 min, followed by subsiding at −40°C overnight and centrifugation at 12,000 rpm at 4°C for 15 min. Next, 80 μl of the supernatant was collected and transferred to a new Eppendorf tube, followed by addition of 40 μl carbonate solution with a concentration of 100 mM and 40 μl benzoyl chloride acetonitrile solution with a concentration of 2% and incubation for 30 min. After adding 10 μl of internal standard to the samples, which were centrifuged at 12,000 rpm for 15 min at 4°C. Next, 40 μl of supernatant was added to 20 μl H_2_O, and then, the samples were removed to an auto-sampler vial for ultra high performance liquid chromatography coupled with mass spectrometry (UHPLC–MS/MS) analyses.

The UHPLC–MS/MS analyses were conducted as previously described (Wong et al., 2016). The UHPLC separations were performed using an ExionLC system equipped with a Waters ACQUITY UPLC HSS T3 (100 mm × 2.1 mm, 1.8 μm). Mobile phase A was formic acid (concentration 0.1%) and ammonium acetate in water (concentration 1 mM), and mobile phase B was acetonitrile. We set the column temperature at 40°C. We set the auto-sampler temperature at 4°C, and each injection volume was 1 μl. We used an AB Sciex QTrap 6,500+ mass spectrometer for analytical development. The classic ion source parameters were set as follows: ion spray voltage: +5,000 V, curtain gas: 35 psi, temperature: 400°C, ion source gas 1 pressure: 60 psi, ion source gas 2 pressure: 60 psi. Multiple reaction monitoring data were processed using Skyline software.

### Correlation analysis of species and metabolites

Spearman correlation was analyzed to assess the association between gut microbiota and differentially expressed metabolites. The top 20 species between the Ctrl and Lop groups and 21 differentially expressed metabolites were also analyzed.

### Data analysis

Defecation-related parameters are expressed as mean values ± standard deviation. If the data conformed to the normal distribution, a one-way ANOVA was used, followed by Bonferroni post-hoc test for multiple comparisons. Different abundances at the phylum, genus, and species levels and functional models among three groups were examined by the Kruskal–Wallis *H*-test. *p* values below 0.05 were considered to represent a significant difference.

## Results

### Phe alleviate defecation-related parameters in constipated rats

To explore the effect of Phe on constipation, rats were pretreated with Lop for 7 days, followed by Phe for another 7 days (Figure 1A). The results showed that Phe intervention significantly inhibited the reduction of body weight of constipated rats (*p* < 0.05, Lop + Phe vs. Lop group; Figure 1B). In addition, the number of feces, wet feces weight, fecal water content, and gastrointestinal transit ratio were dramatically reduced in the Lop group compared with the Ctrl group (*p* < 0.05), but these trends were reversed by Phe intervention (*p* < 0.05, Lop + Phe vs. Lop group; Figures 1C–G). There was no distinction in fecal dry weight between the Lop + Phe and Lop groups (Figure 1E), and we speculated that the decrease in fecal water content was associated with wet weight. To further investigate the effect of Phe on constipation in rats, hematoxylin and eosin staining was used to evaluate the alterations of colon morphology, gut barrier, and inflammatory state. Representative hematoxylin–eosin-stained sections of the colon were shown in Figure 1H. In Lop-treated rats, crypt depth and crypt surface were disrupted, inflammatory cell infiltration was evident, and the thickness of muscle layers was decreased. However, Phe partially restored these changes.

### Phe remold the gut microbial profile In constipated rats

**Figure 2:**
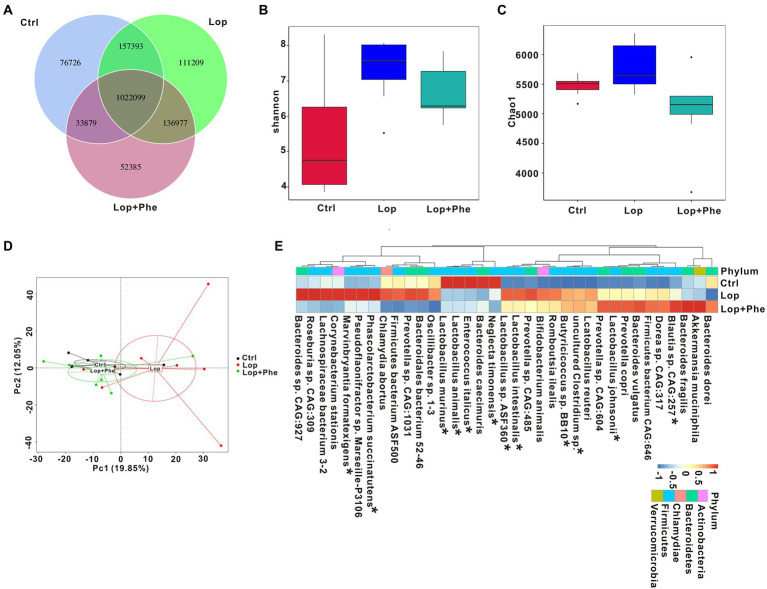
Phe reverses dysbiosis of the gut microbiota in constipated rats, as revealed by metagenomics sequencing data. **(A)** Venn diagram depicting the number of shared (overlap) and unique (non-overlap) genes among the Ctrl, Lop, and Lop + Phe groups. **(B,C)** Alpha-diversity index for the three groups at the species level. Shannon index **(B)** and Chao index **(C)**. **(D)** Beta diversity as determined using PCA plots. **(E)** Heatmap comparison between the three groups at the species level. *p < 0.05, Kruskal–Wallis H test.

To further investigate the relationship between Phe-relieving constipation-related parameters and changes in gut microbial composition and to reveal changes in gut microbial profile and function at the species level, metagenomic sequencing was applied to 7, 8, and 7 fecal samples of the Ctrl, Lop, and Lop + Phe groups, respectively. As shown in Figure 2A, the Ctrl group contained 1,290,097 specific genes, the Lop group contained 1,327,678 specific genes, and the Lop + Phe group contained 1,245,340 specific genes. The Shannon index and the Chao1 index were used to evaluate the alpha-diversity of gut microbiota between groups. As shown in Figures 2B,C, consistent with the specific gene results, the two indices were the highest in the Lop group and were lowest after Phe intervention, but this difference was not statistically significant. PCA at the genus level revealed a significant difference in microbial composition between the Ctrl and Lop groups, but the difference was reduced after Phe intervention (Figure 2D). At the genus level, the relative abundances of *g_Lactobacillus* were 0.335, 0.164, and 0.174 in the Ctrl group, Lop group, and Lop + Phe group, respectively (*p* < 0.05). Among the top 35 species in terms of relative abundance, the *Lactobacillus intestinalis*, *Lactobacillus* sp. *ASF360*, *Phascolarctobacterium succinatutens*, and *Marvinbryantia formatexigens* were significantly higher in the Lop group than in the Ctrl group, and the relative abundances of *Enterococcus italicus*, *Lactobacillus animalis*, and *Lactobacillus murinus* were dramatically lower in the Lop group than in the Ctrl group. However, the changes of these species showed opposite changes after Phe intervention (Kruskal–Wallis H test, Figure 2E).

### Phe alter gut microbial function In constipated rats

**Figure 3:**
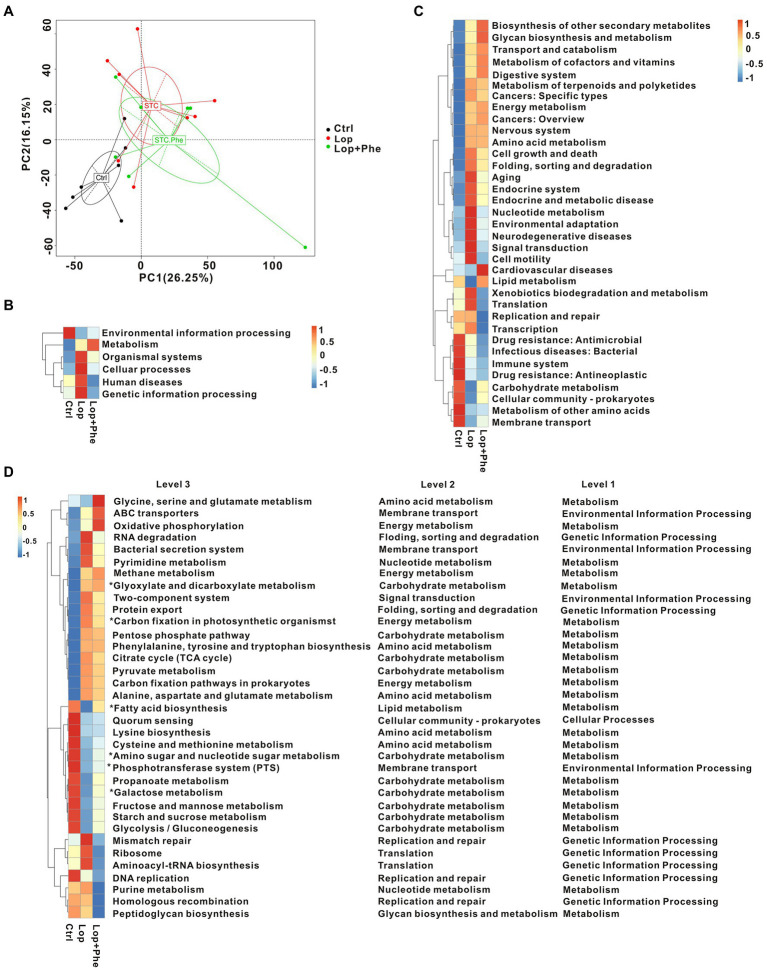
Microbial gene function prediction by KEGG analysis among the three groups. **(A)** PCA based on the Bray–Curtis distance of the KEGG model between the three groups. **(B–D)** Mean abundance of differentially enriched KEGG pathways among the three groups at level 1, level 2, and level 3. *p < 0.05, Kruskal–Wallis H test.

For functional comparison of bacterial genes, we examined gut microbial function in the three groups. A total of 3,863,115 genes were identified in our study. Based on KEGG modules, PCA revealed that the functions of gut microbiota were different among the three groups (Figure 3A). Meanwhile, there were differences in KEGG pathways at level 1 (Figure 3B), level 2 (Figure 3C), and level 3 (Figure 3D), among the three groups. In carbohydrate metabolism, the tricarboxylic acid (TCA) cycle and pyruvate metabolism showed an increasing trend in the metabolic pathways of the Lop group, and the levels decreased after Phe administration. Amino sugar and nucleotide sugar metabolism, propanoate metabolism, galactose metabolism, fructose and mannose metabolism, the starch metabolic pathways, sucrose metabolism, and glycolysis/gluconeogenesis were decreased in the Lop group, and the levels were increased after administration of Phe. These results present a valuable molecular basis for revealing the intestinal dysfunction in constipated patients.

Overall, microbiome-related gene analysis showed that the Lop group exhibited dysfunction in multiple KEGG pathways compared to the Ctrl group, and partial improvement was observed after Phe intervention. Biodegradation of carbohydrates, amino acids, lipids, nucleotides, and xenobiotics was disrupted in the Lop group compared to the Ctrl group. The analysis further showed that the alleviation of constipation symptoms in the Lop + Phe group may partly be related to the effects of Phe on the gut microbiota and metabolites.

### Phe affect metabolic patterns in constipated rats

**Figure 4:**
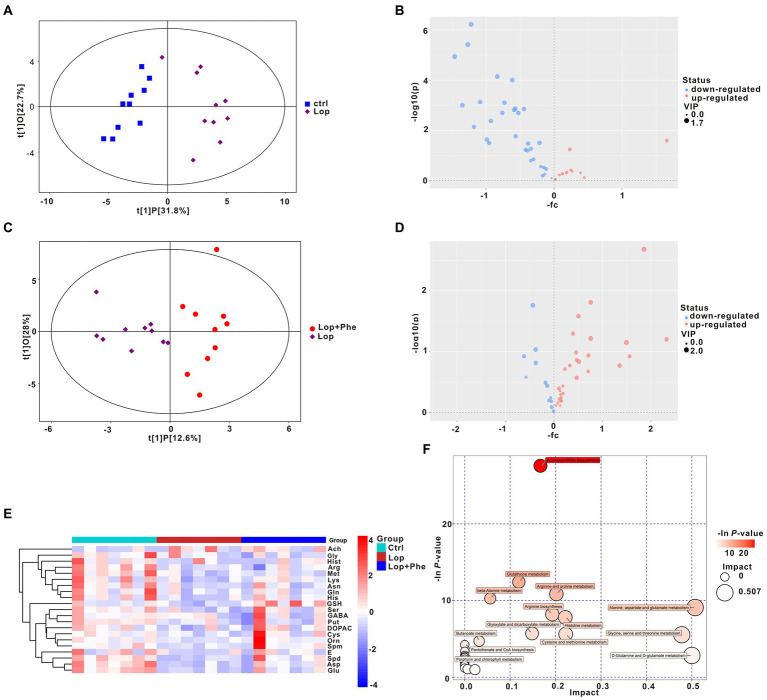
Partial reversal of metabolic disturbances by Phe in constipated rats, as revealed by targeting metabolomic data. **(A,C)** OPLS-DA. **(B,D)** Volcano plot of differential metabolites between the Ctrl and Lop groups **(B)** and between the Lop and Lop + Phe groups **(D)**. **(E)** Heatmap of the 21 differentially abundant metabolites between the three groups. **(F)** Bubble plot of metabolic pathways of differentially abundant metabolites among the three groups.

Microbial-derived metabolites affect the host through multiple pathways. An increasing body of evidence shows that some metabolites of gut microbiota could enter the blood and have an important impact on the host’s physiology and behavior. We previously found through untargeted metabolomics that there were differences in various neurotransmitters in the constipation group compared with the Ctrl group. Therefore, we performed targeted metabolomic analysis of neurotransmitters in 10 Ctrl, 10 Lop, and 10 Lop + Phe serum samples by UHPLC–MS/MS and explored potential relationships between gut microbiota and metabolites. The serum samples from different groups were separated according to orthogonal partial least squares discriminant analysis (OPLS-DA; Figures 4A,C). Moreover, volcano plots and KEGG cluster heatmap results of metabolites also showed differences in metabolism between groups (Figures 4B,D,E), which suggested that there were different metabolic patterns among the three groups.

Specifically, there were differences in 21 of the 42 neurotransmitters detected by targeted metabolomics in the Lop group compared to the Ctrl group, among which Acetylcholine (Ach) levels increased 3.155 times, and the remaining 20 neurotransmitter species showed a decreasing trend (*p* < 0.05; Table 1). After Phe intervention, the increase in Ach levels in the Lop group was partially inhibited, and the decrease in the levels of 18 other metabolites (all except for glycine (Gly) and methionine (Met)) was partially inhibited (Table 2). Further analysis revealed that the levels of L-asparagine (Asn), L-Cysteine (Cys), 3,4-Dihydroxyphenylacetic acid (DOPAC), Epinephrine (E), 4-Aminobutyric acid (GABA), L-glutamine (Gln), L-histidine (His), lysine (Lys), Putrescine (Put), serine (Ser), and Spermidine (Spd) recovered to more than 70% of the Ctrl levels after Phe intervention. In total, 24 KEGG pathways were significantly different among the three groups (Figure 4F). Among them, the top 5 enriched pathways included aminoacyl-tRNA biosynthesis, glutathione metabolism, arginine and proline metabolism, beta-alanine metabolism, and alanine, aspartate (Asp), and glutamate metabolism.

**Table 1:** Differential abundance of metabolites between the Ctrl and Lop groups.

**Compound name**	**Mean Lop**	**Mean Ctrl**	**VIP**	***P*-value**	**Fold change**	**Change in Lop**
Ach	27.772	8.802	0.678	0.026	3.155	up
Arg	12026.360	21460.269	1.486	0.000	0.560	down
Asn	9037.748	13592.558	1.337	0.002	0.665	down
Asp	3529.622	9735.757	1.691	0.000	0.363	down
Cys	49.734	82.565	1.322	0.001	0.602	down
DOPAC	13.281	19.137	1.348	0.002	0.694	down
E	15.214	29.511	1.034	0.032	0.516	down
GABA	119.434	138.503	1.206	0.032	0.862	down
GSH	10.109	22.901	1.034	0.007	0.441	down
Gln	87534.066	129572.276	1.133	0.017	0.676	down
Glu	8874.147	21415.709	1.677	0.000	0.414	down
Gly	68439.814	89230.474	1.184	0.033	0.767	down
His	3878.630	7399.997	1.261	0.004	0.524	down
Hist	293.569	582.285	1.214	0.023	0.504	down
Lys	62604.442	106242.319	1.241	0.002	0.589	down
Met	8050.240	11968.369	1.183	0.001	0.673	down
Orn	1932.103	4109.374	1.300	0.001	0.470	down
Put	188.678	289.053	1.590	0.000	0.653	down
Ser	21767.262	29607.288	1.213	0.001	0.735	down
Spd	194.086	452.374	1.637	0.000	0.429	down
Spm	9.768	24.908	1.519	0.001	0.392	down

**Table 2:** Differential abundance of metabolites between the Lop and Lop + Phe groups.

**Compound name**	**Mean Lop + Phe**	**Mean Lop**	**VIP**	***P*-value**	**Fold change**	**Change in Lop + Phe**
Ach	18.579	27.772	0.331	0.262	0.669	down
Arg	13340.609	12026.360	0.268	0.568	1.109	up
Asn	9528.006	9037.748	0.063	0.701	1.054	up
Asp	5964.661	3529.622	1.706	0.015	1.690	up
Cys	81.309	49.734	1.098	0.115	1.635	up
DOPAC	18.865	13.281	1.522	0.026	1.420	up
E	21.034	15.214	1.250	0.267	1.383	up
GABA	139.805	119.434	0.564	0.193	1.171	up
GSH	36.684	10.109	2.036	0.002	3.629	up
Gln	96163.783	87534.066	0.187	0.506	1.099	up
Glu	12292.390	8874.147	0.879	0.137	1.385	up
Gly	60561.696	68439.814	0.968	0.321	0.885	down
His	5324.379	3878.630	1.024	0.103	1.373	up
Hist	320.637	293.569	0.079	0.765	1.092	up
Lys	82159.691	62604.442	1.054	0.050	1.312	up
Met	7728.632	8050.240	0.592	0.646	0.960	down
Orn	4940.494	1932.103	1.393	0.169	2.557	up
Put	214.913	188.678	0.439	0.484	1.139	up
Ser	24717.766	21767.262	0.330	0.366	1.136	up
Spd	328.355	194.086	1.614	0.061	1.692	up
Spm	27.528	9.768	1.982	0.070	2.818	up

**Figure 5:**
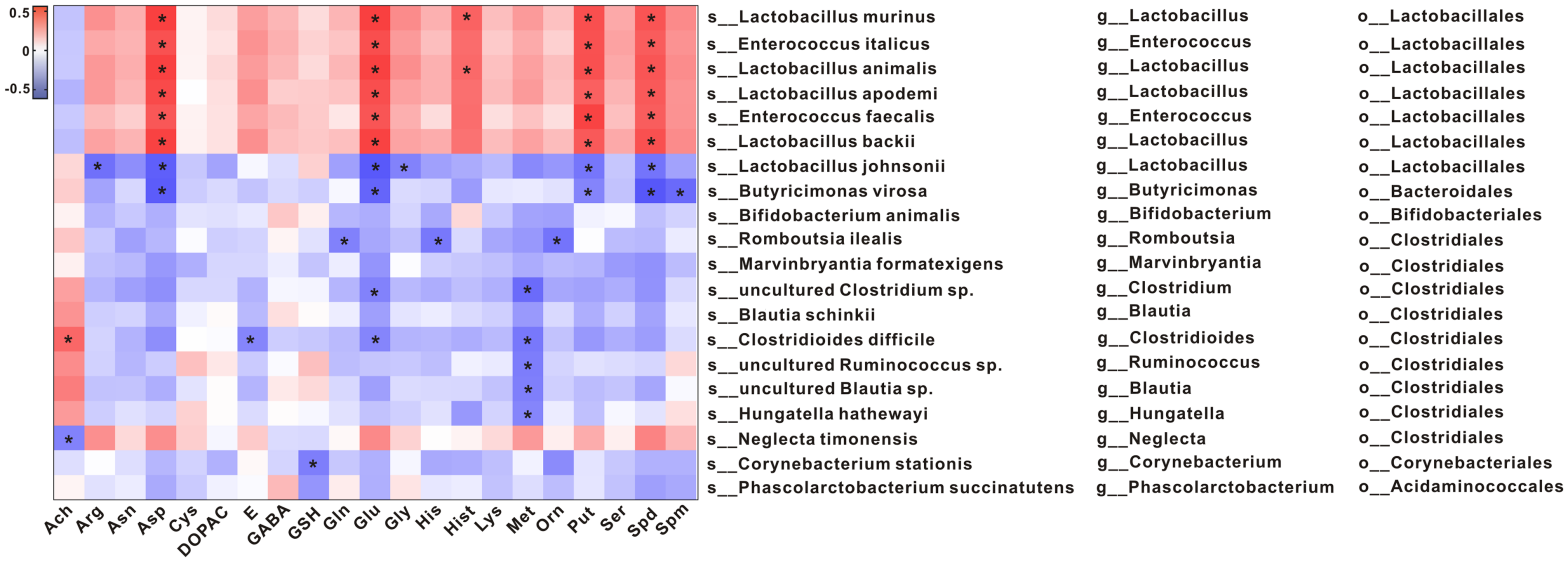
Spearman correlations between metabolites and the gut microbial communities at the species level. *p < 0.05.

To explore potential relationships between changes in gut microbiota and metabolites, a correlation matrix was generated using Spearman correlation. As shown in Figure 5, the abundance of species such as *s_Lactobacillus murinus*, *s_Enterococcus italicus*, *s_Lactobacillus animalis*, *s_Lactobacillus apodemi*, *s_Enterococcus faecalis*, and *s_Lactobacillus backii*, which belong to the same order as *o_Lactobacillales*, was positively correlated with Asn, Glu (L-Glutamic acid), Put, and Spd levels. The abundances of *s_Lactobacillus johnsonii* and *s_Butyricimonas virosa*, which belong to the same order as *o_Lactobacillales* and *o_Bacteroidales*, respectively, were negatively correlated with Asn, Glu, Put, and Spd levels.

## Discussion

In recent years, gut health has received attention, and constipation is one of the most common gastrointestinal diseases (Mugie et al., 2011). Many studies have shown that there is intestinal flora disturbances in patients with constipation, but the results are inconsistent (Ohkusa et al., 2019). This may be due to the interference of constipation by multiple confounding factors such as gender, age, hormone levels, and detection methods. Therefore, it is necessary to control for confounding factors and adopt a multi-omics research strategy to draw more reliable conclusions. Phe is involved in the metabolism of various neurotransmitters, and it acts as a key mediator of host–microbe interactions and participates in host physiological activities (Krautkramer et al., 2021). Our previous study found that the serum Phe level was decreased in women of childbearing age with constipation (Liu et al., 2022). In the present study, metagenomics and targeted metabolomics were combined to explore the changes in gut microbiota and metabolism in constipated rats on the basis of controlling for the gender and age of mice.

As shown in our results, there were differences in the gut microbiota profiles among the three groups. In the present study, Phe reduced the abundance of several harmful bacteria and enriched the abundance of multiple beneficial bacteria at the species level. We found that *g_Lactobacillus* abundance was significantly reduced in constipated rats, which was consistent with the results of Khalif et al., (2005) earlier study in adult patients with functional constipation. However, Zoppi et al., (1998) found that *g*_*Lactobacillus* abundance was significantly elevated in constipated children, which may be connected with the different composition of gut microbiota at different ages, complicating gut microbiota research. Overall, *g_Lactobacillus* was involved in the development of constipation. Furthermore, this study found *Lactobacillus intestinalis* and *Lactobacillus* sp. *ASF360*, which belong to the *g_Lactobacillus*, were significantly higher in the Lop group than in the Ctrl group, the abundances of *L. animalis* and *L. murinus* were lower in the Lop group than in the Ctrl group, but these changes were reversed by Phe intervention. In addition, a randomized double-blind clinical trial confirmed that *Lactobacillus reuteri DSM 17938* was effectived in children with functional constipation (Kubota et al., 2020). However, *Lactobacillus reuteri* was used to treat acute diarrhea in young children (Shornikova et al., 1997). These suggest that some specific *Lactobacillus* species may perform a key role in the pathophysiology of functional constipation.

Food proteins are hydrolyzed in the gastrointestinal tract to produce Phe, which are sensed and handled by the host and gut flora (Liu et al., 2020). Phenylalanine is mainly catabolized by Phe hydroxylase to generate tyrosine (Tyr) in the liver, which is then involved in the synthesis of neurotransmitters such as dopamine and norepinephrine, adrenal hormones, or melanin. Altered Phe and Tyr microbial metabolism is known to play a role in inflammatory bowel disease (Santoru et al., 2017). Untargeted metabolomic studies have shown that increased fecal phenylethylamine (a metabolite of phenylalanine) levels distinguish Crohn’s disease patients from controls (Jacobs et al., 2016; Santoru et al., 2017). However, in our previous study, it was found that the serum Phe level was significantly reduced in Constipated Women of Reproductive Age, and the Phe metabolic pathway was abnormal (Liu et al., 2022), suggesting that it may have different roles in different intestinal diseases, which means that the roles of gut microbiota and metabolites in intestinal diseases are complex. Combined analysis of multi-omics will allow us to better understand the mechanism of action of microbial metabolites in intestinal diseases.

Functional constipation is defined as a disorder of intestinal brain function interaction (Grundy et al., 2006), and is accompanied by anxiety and depression in IBD patients who are mainly constipated (Fond et al., 2014). Therefore, more attention should be paid to the neuropsychiatric research of constipation patients. In this study, 21 levels of 42 neurotransmitters were found to be different through targeted neurotransmitter metabolomics technology. The levels of DOPAC and E, which are related to Phe metabolism, were decreased in the Lop group than in the Ctrl group, but DOPAC returned to near-normal levels after Phe intervention, which may be the reason why we did not find any difference in Phe levels among the three groups in our study. In addition, a previous study found that there was a redox imbalance in patients with functional constipation (Hajji et al., 2020). In this study, we found that the levels of the redox-related metabolites Cys and Glutathione (GSH) were significantly decreased in the Lop group (Table 1). GSH levels were significantly increased after Phe intervention (Table 2). In addition, four neurotransmitters Asn, Glu, Put, and Spd attracted our attention, and they showed obvious positive/negative correlation with differential bacteria.

The abundances of *s_Lactobacillus murinus*, *s_Enterococcus italicus*, *s_Lactobacillus animalis*, *s_Lactobacillus apodemi*, *s_Enterococcus faecalis*, and *s_Lactobacillus backii* were positively correlated with Asn, Glu, Put, and Spd levels. The abundances of *s_Lactobacillus johnsonii* and *s_Butyricimonas virosa* were negatively correlated with Asn, Glu, Put, and Spd levels. A recent study has shown that Asp was important for amino acid homeostasis and was closely related to tumor progression through mTORC1 activity and mitochondrial respiration (Hettmer et al., 2015; Krall et al., 2021). In the present study, we found that the Asp level was significantly decreased in constipated rats and that it recovered to 70% of the normal level after Phe intervention, suggesting that Phe may affect Asp levels and hence cell energy metabolism and proliferation. Glu is the main excitatory neurotransmitter in mammals, which can be metabolized into Gln (Niciu et al., 2012). Gln has been shown to promote intestinal cell proliferation, regulate tight junction proteins, inhibit pro-inflammatory signals, and protect cells from apoptosis and cell stress in normal and pathological processes.A study has shown that Put and Spd derived from gut microbiota can be used as substrates to promote the functional hypusine modification of eIF5A (Hyp-eIF5A). Hyp-eIF5A can promote colonic epithelial cell proliferation, regulate macrophages, and maintain intestinal mucosal homeostasis (Nakamura et al., 2021). In addition, spermidine or L-arginine (Arg) exerts beneficial effects on the intestinal immunity by promoting the differentiation of T cells (Carriche et al., 2021). Epidemiological data show that the increase of dietary Spd intake is related to a decrease in the prevalence of colorectal cancer. In addition to colorectal cancer, spermidine can also regulate colitis (Vargas et al., 2015; Gobert et al., 2022). Spermine oxidase (SMOX) can promote spermidine production. Gobert et al. found that deletion of *SMOX* in mice aggravated dextran sulfate sodium (DSS)-induced colitis and azoxymethane (AOM) /DSS-induced colon tumorigenesis, increased α-defensin expression, and induced gut dysbiosis, while spermidine supplementation reversed these changes (Gobert et al., 2022). In the present study, it was found that the levels of Put and Spd were lower in the Lop group than the Ctrl group and that they were negatively correlated with the abundances of *s_Lactobacillus johnsonii* and *s_Butyricimonas virosa*. Put and Spd may play a key role in functional constipation by regulating intestinal inflammation and intestinal mucosa, which provides a direction for future research.

## Conclusion

The present study demonstrated the beneficial effects of Phe on Lop-induced constipation in rats, as indicated by increased fecal water content, higher intestinal motility, reduced intestinal inflammation, and higher integrity of the intestinal mucosal barrier. In addition, Phe reshaped the gut microbiota and metabolite profiles. Our study demonstrates the potential application of Phe in the treatment of constipation.\.

## Data availability statement

The datasets presented in this study can be found in online repositories. The names of the repository/repositories and accession number(s) can be found below: NCBI PRJNA870741 and Metabolights database (MTBLS5717).

## Ethics statement

The animal study was reviewed and approved by Animal Experiment Committee of the China Institute for Radiation Protection.

## Author contributions

XD and XSu: coordinated the project and conceived the study. CY: participated in the design and execution of the project and prepared and revised the manuscript. XB, TH, XX, XSh, XZ, and TW: collected samples. MZ: performed the statistical analysis. All authors contributed to the article and approved the submitted version.

## Funding

The study was funded by the Shanxi Province “136” Revitalization Medical Project Construction Fund, and the Chinese Nutrition Society Nutrition Science Foundation-ZD Tizhi and Health Fund (CNS-ZD2020-59).

## Conflict of interest

The authors declare that the research was conducted in the absence of any commercial or financial relationships that could be construed as a potential conflict of interest.

## Publisher’s note

All claims expressed in this article are solely those of the authors and do not necessarily represent those of their affiliated organizations, or those of the publisher, the editors and the reviewers. Any product that may be evaluated in this article, or claim that may be made by its manufacturer, is not guaranteed or endorsed by the publisher.
